# Effect of the combined action of *Quercus cortex* extract and probiotic substances on the immunity and productivity of broiler chickens

**DOI:** 10.14202/vetworld.2018.1416-1422

**Published:** 2018-10-13

**Authors:** G. K. Duskaev, S. G. Rakhmatullin, N. M. Kazachkova, Y. V. Sheida, I. N. Mikolaychik, L. A. Morozova, B. H. Galiev

**Affiliations:** 1 Department for Feeding Agricultural Animals and Fodder Technology, Federal Research Centre of Biological Systems and Agro-technologies of the Russian Academy of Sciences, Orenburg - 460 000, Russia; 2 Institute of Bioelements, Orenburg State University, Orenburg, 460018, Russia; 3 Kurgan State Agriculture Academy, Lesnikovo, Ketovsky, Kurgan Region, 641300, Russia

**Keywords:** broiler chickens, growth, plant extract, probiotic

## Abstract

**Aim:**

This study was designed to investigate the synergistic effect of the combined action of probiotic bacterial strains (*Bifidobacterium adolescentis* and *Lactobacillus acidophilus*) and *Quercus cortex* extract as biologically active substances in the feed on the immunity and productivity of *Gallus gallus domesticus*.

**Materials and Methods:**

For the experiment, 120 7-day-old broiler chickens were selected (4 groups, n=30, 3 replicates with 10 birds in each group). The groups were as follows: The reference group - basic diet (BD); experimental Group I - BD + *Q. cortex* extract *(Q. cortex)*, 2.5 ml/kg of body weight; experimental Group II - BD + probiotic preparation based on *B. adolescentis*, 80.0 million colony-forming units (CFU), and *L. acidophilus*, 1.0 million CFU (dosage in accordance with the recommendations of the manufacturer); and experimental Group III - BD + probiotic + extract of *Q. cortex*. The following methods of study were used: Chemiluminescence and biochemical and hematological analysis.

**Results:**

The results of the experiment showed a slight decrease in the level of leukocytes in Groups II (p≤0.05) and III, and of hemoglobin in Group III (p≤0.05), compared to the reference group. The level of alanine aminotransferase and aspartate aminotransferase in Group II was higher than both the reference group (p≤0.05) and the other groups. Introduction of *Q. cortex* extract into the diet increased the level of triglycerides (p≤0.05) and urea in the blood serum. The combined use of probiotic preparations and the extract resulted in an increase in the level of iron in the blood serum by 78.1% (p≤0.05) in Group III. An increase in indicators of the antioxidant system (catalase increased in Group I by 27.2% (p≤0.05) and by 3.0–12.7% in other groups; superoxide dismutase increased by 3.0–13.2%) and nonspecific immunity (β-lysine increased by 8.8–16.0%) was noted. Introduction of the extract and probiotic preparation into the diet contributed to increasing the live weight of chickens at the age of 15 days by 5.9 and 7.4%, respectively (p≤0.05). In experimental Group II, this trend continued, and by the end of the period, the weight of animals exceeded that of their peers in other groups by 0.7-7.0%. Given the high preservation rate of poultry in the II and III Groups, and the low feed consumption per 1 kg of live weight gain (by 3.1–6.7%), the efficiency of growth was higher than in the reference group.

**Conclusion:**

Thus, the combined use of probiotic strains of bacteria and *Q. cortex* extract helped to increase the antioxidant activity of the organism and antimicrobial components of blood plasma compared with broiler chickens with similar growth rates but without the supplementation of this combination.

## Introduction

The current trend in animal nutrition is feeding with more “natural” substances as the use of some additives, such as antibiotic drugs in animal feed, is not allowed by law in many countries due to the development of antibiotic-resistant bacteria [1]. This created an increased interest in the poultry industry aimed at finding other alternatives that may be safe and accepted by the consumers. Lately, probiotics, prebiotics, herbs, spices, or botanical substances (e.g., essential oils) have been considered as good substitutes [2]. Herbs and trees contain various antioxidants with high potential for protecting nutrients from oxidation in the digestive tract in the process of metabolism and can help develop immunity and contribute to the growth of animals [3]. Some medical effects of medicinal plants are related to their secondary metabolites, such as phenols, essential oils, and saponins [4]. Many herbs have a long history of use, even prehistoric use, in preventing or treating diseases of humans and animals due to their availability, ease of use, and the absence of side effects. However, the results of studying the use of herbal mixtures in the diets of broiler chickens have thus far yielded inconsistent results. Some authors claimed a positive effect on broilers’ performance [5], while others noted no effect on weight gain, feed consumption, or feed conversion [6]. One of the studied plants is *Quercus*; this genus has been found to have antioxidant, antifungal, antibacterial, and antitumor activities [7]. The composition of polar fractions of leaves, bark, wood, and galls shows antibacterial and anti-inflammatory activity [8-10], which explains their ethnopharmacological use. Ethanol extract of *Q. leucotrichophora* showed strong antimicrobial activity against *Staphylococcus aureus, Pseudomonas auroginosa, Bacillus subtilis*, and *Escherichia coli* [11]. Physiochemical composition analyses have shown that plants belonging to genus *Quercus* are rich in lignins, hydrolyzable tannins, ellagitannins, flavane ellagitannins, catechins, flavones and proanthocyanidin glycosides, flavonoids, and simple phenols and proanthocyanidin glycosides [12,13]. Probiotics, due to their multipurpose actions, improve animal health and increase the efficiency of poultry production. The interest in using probiotics for poultry has been increasing since animal growth promoters were prohibited in the EU and the associated increased occurrence rate of intestinal infections in poultry mostly caused by *Clostridium perfringens*. From the literature, it is known that probiotics have a positive effect on the immunity of broilers, including when they are combined with substances or parts of plants [14,15]. Immunity indices were maintained at the same level after using strains of *Bacillus* [16]; *Lactobacillus reuteri* increases the growth rates of birds at an early age, stimulates the immune response, and reduces the amount of *E. coli* [17]. Other probiotics do not adversely affect the immunity of birds [18].

Using the selected strains of *Lactobacillus* as feed additives for poultry may result in similar effects with the effect of growth promoters of antibiotics, resulting in weight gain and better feeding efficiency, and resistance to pathogenic bacteria, such as *Salmonella* sp. [19], *C. perfringens* [20,21], *E. coli* [22], or *Campylobacter* sp. [23]. Furthermore, adding strains of *Lactobacillus* into the diet of broilers reduces general fat accumulation [24] and increases size, carcass quality, and egg production [25].

It is assumed that the useful substances of the plant described above, combined with the probiotic, could be used as a feed additive in the broiler diet and to improve growth and carcass quality, stimulate the immune system, and reduce the perceived risk to health. This study aimed to assess the synergistic effect of the combined use of *Quercus cortex* extract and probiotic substances on immunity and productivity in broiler chickens.

## Materials and Methods

### Ethical approval

Poultry maintenance and procedures during the experiments met the requirements of the instructions and recommendations of Russian regulations (order of the Ministry of Health of the USSR No. 755 of 12.08.1977) and “The Guide for Care and Use of Laboratory Animals (National Academy Press, Washington, D.C., 1996).” Every effort has been made to minimize the suffering of the animals and to reduce the number of samples used.

### Preparation of Q. cortex extract

The preparation of *Q. cortex* extract included the following steps: Weighing 50 g amounts of shredded bark (medicinal form), placing the bark into heat-proof ware with 500 ml of hot (70°C) distilled water, and heated in a water bath (30 min) followed by percolation and filtering (deashed filters “White Ribbon,” d 70 mm APEXLAB).

### Broilers, management, and dietary treatments

The research was performed at the center of collective use of scientific equipment of the Federal Research Centre of Biological Systems and Agro-technologies of the Russian Academy of Sciences on “Smena-8” broiler chickens. Acclimatization was conducted for 6 days from day 0 to day 6, and each group was maintained in a 60 cm×50 cm×50 cm box equipped with an incandescent lamp to keep the temperature warm. Feeding began at the age of day 7 and continued to day 42. For the experiment, 120 7-day-old broiler chickens were selected (4 groups, n=30, 3 replicates with 10 birds in each group). The groups were as follows: The reference group - basic diet (BD) (Table-1); experimental Group I - BD + *Q. cortex* extract *(Q. cortex*), 2.5 ml/kg of body weight; experimental Group II - BD + probiotic preparation based on *Bifidobacterium adolescentis -* 80.0 million colony-forming units (CFU), and *Lactobacillus acidophilus* – 1.0 million CFU (dosage in accordance with the recommendations of the manufacturer); and experimental Group III - BD + probiotic + extract of *Q. cortex*.

**Table-1:** Ingredients and nutrient level of basal diets.

Attributes	Starter (7-28 days)	Finisher (29-42 days)
	
	Controls I, II, and III	Controls I, II, and III
Ingredient composition (%)		
Wheat	49.0	43.0
Barley	3.1	0.4
Corn	8.0	25.0
Soybean meal (46% CP)	23.0	17.0
Sunflower meal (38% CP)	5.0	10.0
Sunflower oil	5.0	5.0
Di-calcium phosphate	1.6	1.4
Mel stern	0.9	1.5
Limestone	0.5	0.3
Salt	0.32	0.22
DL-methionine	0.18	0.16
L-Lysine	0.35	0.17
Vitamin-mineral premixaa	2.0	2.0
Calculated nutrients metabolizable energy (kkal/100 g)	296.0	302.0
Crude protein	24.0	19.1
Methionine+cysteine	0.87	0.79
Lysine	1.35	0.96
Calcium	0.95	1.0
Available phosphorus	0.54	0.48

^a^Supplied following per kilogram of diet: Vitamin A=7,000 IU, Vitamin D3=800 IU, Vitamin E=9 IU, Vitamin K3=1.1 mg, Thiamine: 0.7 mg, Riboflavin=3.0 mg, Vitamin B6=1 mg, Vitamin B12=0.01 mg, Vitamin C=50 mg, Mn=23 mg, Fe=17 mg, Zn=11 mg, Cu=2.5 mg, I=0.4 mg, Se=0.2 mg

Formulation of diets for the experimental poultry was based on the recommendations of VNITIP (The All-Russian Research and Technological Institute of Poultry). The microclimate in the room was consistent with the VNITIP recommendations and requirements [26]. The growth was monitored every day by individual weighing. Experimental poultry was fed twice a day; the consumption was recorded every day, *Q. cortex* extract was supplied in the drinking water. Poultry was decapitated under Nembutal ether on the 42^nd^ day.

### Sampling and analytical procedures

Blood sampling was taken from the brown brachialis vein by as much as 1 cc. Blood samples for the study were placed in sterile vacuum tubes with anticoagulant EDTA and vacuum sterile tubes to produce blood serum.

The morphological indicators of blood were determined with the use of an automatic hematology analyzer URIT-2900 Vet Plus (Medial URIT Electronic Co., China). Biochemical analysis of blood serum was performed on the automatic biochemical analyzer CS-T240 (“Dirui Industrial Co., Ltd”, China) with the use of commercially available biochemical veterinary kits DiaBet Test (Russia) and commercially available biochemical kits Randox Laboratories Limited, (UK). The following parameters were studied: Erythrocytes, platelets, leukocytes, lymphocytes, monocytes, granulocytes, hemoglobin, glucose, total protein, albumin, urea, bilirubin, cholesterol, triglycerides, creatinine, calcium, phosphorus, iron, alanine aminotransferase (ALT), aspartate aminotransferase (AST), gamma-glutamyl transpeptidase, lactate dehydrogenase (LDH), superoxide dismutase (SOD), lysozyme activity of blood serum (LASK), and β-lysines.

Serum was studied no later than 2 h after sampling. The total amount of antioxidants was determined by measuring chemiluminescence of blood serum samples in the presence of luminol and hydrogen peroxide. During the experiments, indicators of natural resistance were determined: Activity of lysozyme, according to Ermolieva and Buyanovsky [27], and activity of β-lysines by the accelerated method of Bukharin [27].

### Statistical analysis

Statistical analysis was performed usingSPSS Statistics Version 20 (IBM, USA) program, which was used to calculate the average value (M), standard deviation error (m). Further analysis used the Wilcoxon signed-rank test, and results were considered significant with a level of 95% (p≤0.05).

## Results and Discussion

The values of hematological parameters obtained during this research were within the normal ranges as reported by Anon [28], indicating that poultry was adequately fed and therefore not anemic or showed no signs of diseases or parasitic problems (Table-2). The number of blood corpuscles in experimental Group III was lower than those in other groups, lower the level of hemoglobin by 12.8% (p≤0.05) compared to the reference group. These results differ from those obtained earlier; there are data on the lack of influence of probiotic drugs on the blood elements in the literature [29,30]. At the same time, it must be taken into account that we used other strains of probiotic bacteria.

**Table-2:** Morphological and biochemical parameters of broiler chicken blood.

Index	Group			

	Control	I	II	III
Red blood cells, 10^12^/l	2.06±0.14	1.97±0.07	1.93±0.08	1.83±0.07
Thrombocytes 10^9^/l	67.50±7.35	68.00±2.38	63.25±2.78	65.60±1.57
Hemoglobin, g/l	131.25±9.54	127.75±4.96	124.25±6.55	114.40±4.47^a^
Glucose, mmol/l	10.68±0.27	10.40±0.10	10.62±0.34	10.50±0.11
Total protein, g/l	32.25±1.62	33.86±0.29	33.04±1.16	32.06±0.47
Albumin, g/l	13.25±0.75	14.25±0.25	14.00±0.71	13.40±0.40
Urea, mmol/l	0.88±0.03	0.93±0.03	0.90±0.07	0.90±0.05
Direct bilirubin, µmol/l	0.41±0.02	0.45±0.03	0.43±0.04	0.42±0.04
Cholesterol mmol/l	3.95±0.12	3.96±0.10	3.95±0.11	3.75±0.20
Triglycerides, mmol/l	0.07±0.01	0.03±0.02^a^	0.05±0.02	0.03±0.01^a^
Creatinine, µmol/l	70.65±8.13	71.13±4.48	77.18±10.49	84.94±12.14
Calcium, mmol/l	2.53±0.06	2.68±0.04	2.45±0.06	2.54±0.08
Phosphorus, mmol/l	2.07±0.28	1.75±0.17	2.59±0.85	2.02±0.75
Iron, µmol/l	28.8±0.80	32.83±5.07	37.50±3.05	51.32±6.50^a^

a p≤0.05 in comparison with the control group

Analysis of the data showed a decreased level of glucose and a decreased concentration of triglycerides in the blood of poultry in the experimental groups (Table-2), which might be due to the beneficial effect of the substances in the composition of the medicinal plants. Some flavonoids have insulin-like action and thus can reduce the level of glucose in the blood [31].

A decreased level of glucose in the blood of broilers with the introduction of tannic acid into the diet was observed in earlier studies [32], as well as triglycerides on feeding an essential oil mix obtained from plants [33]. It is known that triglycerides are important energy products, especially those used by chickens for growth [34,35]. In addition, a recent study [36] found that bifidobacteria inhibit fat accumulation, improve insulin resistance, and reduce blood glucose levels in the blood of laboratory animals.

High creatinine content was found in experimental Group III, exceeding the value in the reference group by 20.2% and exceeding the value of experimental Group I by 19.4% and experimental Group II by 10.1%. Creatinine is another important indicator of protein metabolism as it is a by-product of phosphocreatine decomposition in skeletal muscles. Its concentration is directly proportional to the muscle weight associated with age, physical activity, and like most components of blood chemistry, which depend on the diet [37].

The indicators of mineral metabolism were within the norm in all examined chickens. The content of Ca in the group, with the introduction of *Q. cortex* extract into the diet, was higher than that of the reference group by 5.9% - however, the content of P in this group, relative to the reference, was lower than the reference by 15.4%. A remarkable increase in the level of Fe in the three experimental groups was observed - 13.9%, 30.2%, and 78.1%, respectively, compared to the reference group. Although there was the tendency for a decrease in the number of erythrocytes in these groups, the probability of increasing the level of iron due to their destruction was insignificant, as it was known that tannic acid mitigated hepatotoxicity caused by iron [38], while probiotic strains could bind iron [39]. The obtained data contradict the information obtained previously [40], where feeding of grape seed extracts marked a decrease in iron and other minerals in the blood plasma of broilers.

The values of leukocytes in experimental Group I were higher by 15.6% compared to the reference group and by 40.8% and 35.3% compared to the other two experimental groups, respectively (Table-3).

**Table-3:** The content of the white blood cells in broilers of cross “Smena 8” in the context of introducing *Q. cortex* extract.

Index	Group			

	Control	I	II	III
Leukocytes, 10^9^/l	19.9±6.21	23.00±4.51	16.33±2.38	17.00±1.49
Percentage of lymphocytes	85.95±1.17	81.05±1.10	85.53±0.64	88.68±0.69
Percentage of monocytes	6.98±0.48	8.23±0.23^a^	6.80±0.42	5.86±0.31
Percentage of granulocytes	7.08±0.78	10.73±0.89	7.68±0.38	5.46±0.39

a p≤0.05 in comparison with control group. *Q. cortex: Quercus cortex*

This fact is in line with the previous studies performed with the use of thyme extract, which did not reveal any significant increase in the leukocyte count but improved the immunological response of broilers organisms [41]. The combination of the extract and the probiotic strain promotes indicators alignment with the reference group, which confirms the opinion that searching for synergistic substances is required for creating efficient feed additives [42]. The increase in lymphocytes in this group is confirmed by earlier conclusions [43] that bifidobacteria promote the activation of these cells in animals. The monocyte count in experimental Group I was higher by 17.9% compared to the reference group.

The levels of AST, ALT, LDH, and catalase activity were within the norm; however, we note a tendency for these values to fluctuate in the experimental groups (Table-4).

**Table-4:** Activity of blood serum enzymes and indicators of non-specific immunity in broilers of cross “Smena 8” in the context *Q. cortex* extract.

Index	Group			

	Control	I	II	III
ALT, units/l	3.8±0.76	4.0±0.36	4.75±0.62^a^	4.08±0.68
AST, units/l	228.8±21.84	219.6±12.53	262.3±32.35^a^	227.32±10.85
g-GT, units/l	16.5±1.04	18.3±2.85	18.50±0.50	19.00±1.82
LDG, units/l	14.2±3.68	14.0±5.57	11.25±3.33	14.80±2.78
SOD, %	879.8±54.2	911.8±38.0	995.9±52.4	906.5±22.9
Catalase, µm H_2_O_2_ l/per/min	1,494.4±63.0	1,901.0±53.3^a^	1,685.3±55.4	1,537.3±51.8
BSLA, %	47.1±0.39	45.3±0.91	48.9±1.2	45.1±0.55
β-lysines, %	72.9±0.40	79.3±0.80	84.6±0.52^a^	80.5±0,51

a p≤0.05 in comparison with control group. ALT=Alanine aminotransferase; AST=Aspartate aminotransferase; g-GT=Gamma-glutamyl transpeptidase; LDH=Lactate dehydrogenase; SOD=Superoxide dismutase; BSLA=Lysozyme activity of blood serum, *Q. cortex: Quercus cortex*

Thus, the ALT activity was remarkably high in experimental Group II; the value of this indicator in this group was higher by 25%, 18.7%, and 16.4%, compared to the reference group, experimental Groups I and III, respectively. The AST activity was also remarkably higher in experimental Group II, exceeding this indicator in the reference group by 16.4%. The LDG activity in all groups was approximately the same; however, there was a decrease in this indicator in experimental Group II by 20.7%, compared to the reference group.

A higher level of catalase was noted in experimental Group I; it exceeded the levels of the reference group by 27.2% (p≤0.05). An increased antioxidant activity on feeding with plant extracts was also noted by other researchers [44]. The introduction of the probiotic preparation into the diet influences activation of β-lysine in blood serum; this indicator increases by 16.1% (p≤0.05), compared to the reference group. This fact is consistent with the results of previous studies [45].

The use of *Q. cortex* extract and the probiotic preparation in the diet influenced the poultry growth rates (Table-5).

**Table-5:** Weekly broiler chicken weight gain, g/head.

Age, days	Group			

	Control	I^a^	II	III
8	115.20±6.2	114.60±9.1	115.60±7.4	114.20±8.5
15	299.50±15.4	317.20±22.5	321.60±21.7^a^	300.40±22.9
22	652.50±28.9	618.80±43.5	684.80±44.0	638.80±32.3
29	1164.00±61.5	1134.40±47.2	1217.20±80.9	1138.80±54.7
36	1787.00±82.2	1660.00±53.5	1797.60±56.0	1682.80±68.7
42	2284.50±86.4	2149.60±87.5	2300.50±60.6	2196.80±94.2

a p≤0.05 in comparison with the control group

Thus, an increase in the live weight of 15-day-old chickens in experimental Groups I and II by 5.9% and 7.4% (p≤0.05) compared to the reference groups, respectively, was noted. This superiority was visible until the end of the accounting period, in which broilers in experimental Group II were superior to their peers in experimental Group I by 7% and in experimental Group III by 4.7%. The cost of feed over the entire period of the experiment in the three experimental groups was lower than in the reference by 13.9%, 5.8%, and 6.8%, respectively. A decreased feed consumption per 1 kg of live weight gain was noted in all experimental groups. Thus, this indicator in broiler chickens from experimental Groups I, II, and III was lower by 3.6%, 6.7%, and 3.1%, respectively, compared to the reference group. This result was consistent with earlier works that indicated an increase in feed consumption [46] and improvements in digestion [47] in cases of feeding poultry using diets prepared with the introduction of herbs and various plant extracts. Herbs and their extracts are known to increase the nutrient requirements of animals [48] due to the complex composition of their constituent biologically active substances, as well as due to the influence of several unknown factors that have a stimulating effect on digestive enzymes [49]. There is possible synergy between combinations of substances [50-52].

## Conclusion

According to the present study, it can be concluded that the synergistic effect of the combined use of *Q. cortex* extract and the probiotic preparation (based on *B. adolescentis* and *L. acidophilus*) in the feeding of broiler chickens is manifested in terms of improvement of immune responses and antioxidant activity of organisms compared with broiler chickens with similar growth rates but without the supplementation of this combination.

## Authors’ Contributions

GKD and SGR equally designed the experiment. NMK, YVS, INM, LAM, and BHG contributed equally to the experimentation. GKD and SGR wrote and edited the article. All authors read and approved the final manuscript.
